# Role of Oxidative Stress in Stem, Cancer, and Cancer Stem Cells

**DOI:** 10.3390/cancers2020859

**Published:** 2010-05-17

**Authors:** Ahmed Abdal Dayem, Hye-Yeon Choi, Jung-Hyun Kim, Ssang-Goo Cho

**Affiliations:** Department of Animal Biotechnology (BK21), RCTCP, and Animal Resources Research Center, Konkuk University, Seoul 143-701, Korea; E-Mails: ahmed_morsy86@yahoo.com (A.A.D.); hyeon@konkuk.ac.kr (H.-Y.C.); nataskjh@konkuk.ac.kr (J.-H.K.)

**Keywords:** oxidative stress, ROS, cancer stem cells, MAPKs, NF-κB, apoptosis, autophagy

## Abstract

The term ‘‘oxidative stress” refers to a cell’s state characterized by excessive production of reactive oxygen species (ROS) and oxidative stress is one of the most important regulatory mechanisms for stem, cancer, and cancer stem cells. The concept of cancer stem cells arose from observations of similarities between the self-renewal mechanism of stem cells and that of cancer stem cells, but compared to normal stem cells, they are believed to have no control over the cell number. ROS have been implicated in diverse processes in various cancers, and generally the increase of ROS in cancer cells is known to play an important role in the initiation and progression of cancer. Additionally, ROS have been considered as the most significant mutagens in stem cells; when elevated, blocking self-renewal and at the same time, serving as a signal stimulating stem cell differentiation. Several signaling pathways enhanced by oxidative stress are suggested to have important roles in tumorigenesis of cancer or cancer stem cells and the self-renewal ability of stem or cancer stem cells. It is now well established that mitochondria play a prominent role in apoptosis and increasing evidence supports that apoptosis and autophagy are physiological phenomena closely linked with oxidative stress. This review elucidates the effect and the mechanism of the oxidative stress on the regulation of stem, cancer, and cancer stem cells and focuses on the cell signaling cascades stimulated by oxidative stress and their mechanism in cancer stem cell formation, as very little is known about the redox status in cancer stem cells. Moreover, we explain the link between ROS and both of apoptosis and autophagy and the impact on cancer development and treatment. Better understanding of this intricate link may shed light on mechanisms that lead to better modes of cancer treatment.

## 1. Introduction

**Figure 1:**
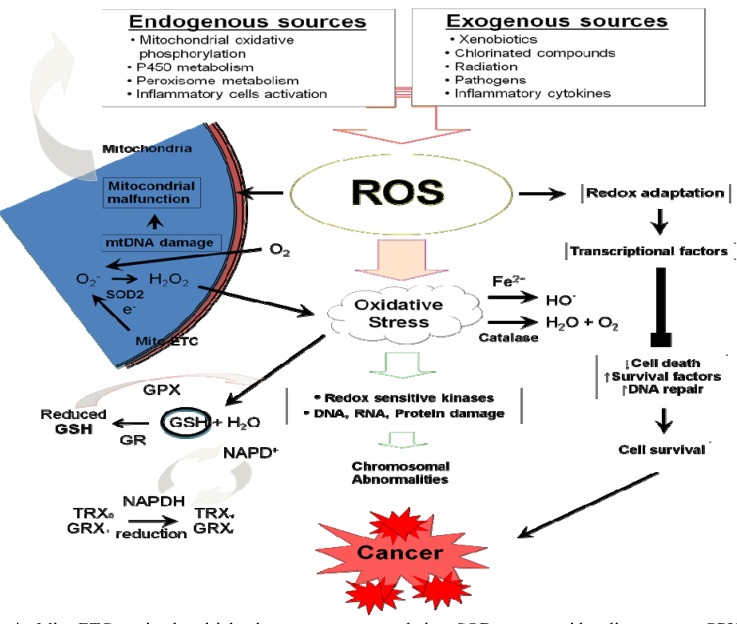
The signaling pathways induced by oxidative stress.

Oxidative stress is defined as a disturbance in the equilibrium between free radicals (FR), reactive oxygen species (ROS), and endogenous antioxidant defense mechanisms [1], or more simply, it is a disturbance in the balance between oxidant-antioxidant states, favoring the oxidant environment [2]. Both of the oxidant and antioxidant species are very important for normal metabolism, signal transduction and regulation of cellular functions. Therefore, each cell in the human body maintains a condition of homeostasis between the oxidant and antioxidant species [3]. Oxidative stress can result in injury to all the important cellular components like proteins, DNA and membrane lipids, which can cause cell death. Oxidative stress has been demonstrated to be involved in various physiological and pathological processes, including DNA damage, proliferation, cell adhesion, and survival. Moreover, there are several experimental and clinical data providing compelling evidence for the involvement of oxidative stress in large number of pathological states including carcinogenesis [4]. The broad definition of the ROS is oxygen-containing, reactive chemical species. Up to 1–3% of the pulmonary intake of oxygen by humans is converted into ROS [5]. There are two ROS subgroups; free radicals such as superoxide radicals (O_2_^.−^) and non-radical ROS such as hydrogen peroxide (H_2_O_2_). Both radicals and non-radical ROS are common in the presence of an oxygen atom, which differentiates them from the reactive nitrogen species (RNS). ROS can be found in the environment, such as in pollutants, tobacco smoke, iron salts, and radiation, or can be generated inside cells, and this can happens through multiple mechanisms. Generally, mitochondria are the most important source of cellular ROS where continuous production of ROS takes place. This is the result of the electron transport chain located in the mitochondrial membrane, which is essential for the energy production inside the cell [6,7]. Additionally, some cytochrome 450 enzymes are also known to produce ROS [8]. The biological functions of ROS and their potential roles in cancer development and disease progression have been investigated for several decades [9]. There are complex interactions between ROS generation, ROS signaling, ROS-induced damage, and carcinogenesis. Figure 1 depicts ROS regulatory pathways, showing how ROS is controlled in cells and a variety aspect of signaling pathways induced by oxidative stress. ROS can directly produce single or double-stranded DNA breaks and cross-links. Prolonged DNA damage leads to serious problems such as induction of signal transduction pathways, arrest or induction of transcription, replication errors, and genomic instability, all of which lead to carcinogenesis [10]. Very little is known about the redox status in cancer stem cells [11]. Therefore, we will focus in this review on the effect of oxidative stress on cancer and stem cells, as understanding redox control in stem and cancer cells may perhaps provide insights into the redox biology of cancer stem cells and development of a new therapeutic strategy [11]. 

* Mito-ETC: mitochondrial electron transport chain, SOD: superoxide dismutases; GPX: glutathione peroxidase; GR: glutathione reductase; GR: glutathione reductase; GRXo, glutaredoxin (oxidized); GRXr: glutaredoxin (reduced); GSHr: glutathione (reduced); TRXo, thioredoxin (oxidized); TRXr: thioredoxin (reduced).

## 2. Stem Cells

Stem cells differ from other kinds of cells in the body. Their unique nature comes from their proliferative capacity and their ability to become specialized. Until recent time, scientists mainly worked with two kinds of stem cells from animals and humans: embryonic stem cells and non-embryonic somatic or adult stem cells.

### 2.1. Embryonic Stem Cells (ESCs)

As their name suggests, embryonic stem cells *(*ESCs) are derived from embryos. ESCs result from the first five or six divisions of the fertilized egg. ESCs are derived from a group of cells called inner cell mass (ICM), which is part of the early (four to five day) embryo called the blastocyst. The progeny of ESCs are the precursors for all of the cells of the adult organs. ESCs are pluripotent; they can produce cell types derived from all three embryonic germ layers. To date, all of the ESC lines generated are pluripotent [12].

### 2.2. Adult Stem Cells (ASCs)

Adult stem cells (ASCs) are thought to be undifferentiated cells, found among differentiated cells in a tissue or organ, which have a self-renewal property and can differentiate into all of the major specialized cell types of the tissue or organ. The sources of ASCs are bone marrow, blood, the cornea and the retina of the eye, brain, skeletal muscle, dental pulp, liver, skin, the lining of the gastrointestinal tract, and the pancreas [13]. ASCs have a limited potential, and they produce cells that differentiate into mature functioning cells and that are responsible for normal tissue renewal. The primary function of the adult stem cells is to maintain the steady state functioning of a cell—called homeostasis—and, within limitations, to replace cells that die because of injury or disease.

## 3. Cancer Cells

The term cancer indicates more than the various types of the disease. Almost every tissue in the body can spawn malignancies. Cancer cells have an insidious property to migrate from their original site and form new masses at distant sites of the body. The activation of proto-oncogenes into oncogenes may contribute to malignancy. Moreover, mutations can contributeto the conversion of proto-oncogenes into carcinogenic oncogenes [14,15]. Basically, cancers originate from normal cells that gain the ability to proliferate abnormally and finally turn malignant. Afterwards, these malignant cells grow clonally into tumors and finally have the ability to metastasize. From the histological point of view, cancer is similar to the tissue of origin. Accordingly, a tumor can be viewed as a dysfunctional organ system [16].

## 4. Cancer Stem Cells (CSCs)

Cancer recurrence after chemotherapy or radiotherapy is initiated by a subpopulation of residual malignant cells that are highly resistant to drug treatment and are believed to be cancer stem cells (CSCs) [16,17]. Of note, a small subpopulation of primary cancer cells expressing stem cell markers was resistant to certain chemotherapeutic agents and radiation [18,19]. The CSCs hypothesis suggests that many cancers are maintained in an organization of rare, slowly dividing “tumor-initiating cells” and rapidly dividing cells [20]. The CSCs are capable of self-renewal and can undergo differentiation to generate the phenotypic heterogeneity observed in tumors. Recently, the defined CSCs have been identified in hematologic, brain, breast, prostate, liver, pancreas, and colon cancers. CSCs are not only the source of the tumor, but also may be responsible for tumor progression [20], metastasis [21], resistance to therapy, and subsequent tumor recurrence [22]. In regard to the significant contribution of redox adaptation in drug resistance, one could speculate that the highly drug-resistant CSC population might use redox regulatory mechanisms to promote cell survival and tolerance to anticancer agents. 

It is worth noting that there are three key observations that classically define the existence of a CSCs population: (1) Within each tumor, only minor populations of cancer cells usually have a tumorigenic potential when transplanted into immuno-deficient mice; (2) One of the important characteristics of the tumorigenic cancer cells is the presence of a unique profile of surface markers, which allows them to be isolated from non-tumorigenic ones by means of flow-cytometry or other immuno-selection procedures; (3) Tumors originating from tumorigenic cells contain mixed populations of tumorigenic and non-tumorigenic cancer cells. As a consequence, they give rise to the full phenotypic heterogeneity of the parent tumor. Accordingly, the term CSC represents a working definition with an operational significance: this term is used to indicate a tumor-initiating cell subset that can give rise to a heterogeneous progeny, similar in composition to the tissue of origin. 

The CSC concept answered many raised but unsolved questions [23]. The CSC concept arose from observation of similarities between the self-renewal mechanism of stem cells and those of cancer cells. The CSCs not only have the capacities of self-renewal and multi-lineage differentiation, but are also similarly surrounded by a specialized cell microenvironment, termed the stem cell niche [24,25]. On the other hand, CSCs are believed to have no control over the cell numbers. 

Both normal stem cells and cancer stem cells are common in several aspects such as:

(1) Self-renewal ability (Asymmetric divisions): This property contributes toward developing a critical mass of cells. Moreover, it generates a quiescent stem cell and a committed progenitor [21];
(2) Self-renewal regulation: Control of the self-renewal ability occurs by similar signaling pathways such as, Wnt, Sonic Hedgehog, Notch, and Polycomb genes (*BMI-1* and *EZH2*);
(3) Telomeres and telomerase activity: This telomerase activity increases the cellular life span. Both have extended telomeres and telomerase activity;
(4) ATP-binding cassette (ABC) transporters: Both express the ABC transporters, which are implicated in the cellular resistance against specific growth-inhibitory drugs;
(5) Surface receptor expression: Both express similar surface receptors such as, c-kit, c-met, LIF-R, CD133, and CXCR4. These surface receptors were identified as stem cell markers or associated with metastasis;
(6) Longevity (Long life span): Both are long-lived;
(7) Resistance to deleterious agents: Both are resistant to deleterious agents;
(8) Metastasis: Both have the metastatic property;
(9) Tumor suppressors: Tumor suppressors, such as p53, p16INK4a, and p19ARF inhibit cancer cell proliferation and stem cell self-renewal [26,27].

Therefore, because of all the forementioned similarities between the normal and the cancer stem cells, it is reasonable to assume that they share some common molecular mechanisms that regulate this critical stem cell function. There are several signaling pathways that have been implicated in both cancer and stem cells [28]. For example, molecular pathways, which play a critical role in controlling stem cell self-renewal, are often deregulated in a number of tumors [29]. PTEN/PI3K/mTOR/STAT3 signaling forms a complex signaling network which maintains the cancer stem cell population within the whole cell population [30]. Recently, there are some therapeutic approaches proposed for the elimination of CSCs by targeting signaling pathways required for the maintenance of self-renewal and differentiation capacities. Various studies have been conducted to investigate the signaling pathways important in the regulation of stem and cancer cells and the role they may have in CSCs. Table 1 summarizes the differences in the signaling pathways among cancer cells, stem cells, and CSCs. Table 2 highlights the differences between normal stem cells and CSCs. 

**Table 1:** The differences among the cancer cells, cancer stem cells, and normal stem cells in the signaling pathways.

Signal pathway	Normal stem cells	Cancer and cancer stem cells
**Polycomb-group protein family (Bmi-1)**	➢ Self-renewal in both hematopoietic and neural stem cells [154]	➢ Leukemic stem cells (LSCs) self- renewal by suppression of the Ink4a/AR Flocus [155]. ➢ Highly expressed in acute myeloid leukemia patients [156,157] as it is essential for the LSC self-renewal.
**Notch**	➢ Neural stem cell expansion regulation *in vivo* and *in vitro* [158]. ➢ Notch targets genes activation, which is involved in T-cell differentiation and self-renewal [159].	➢ Notch signaling pathway mutations result in T-cell acute lymphopblastic leukemia (T-ALL) [122].
**Wnt/β-catenin**	➢ Self-renewal [160]. ➢ HoxB4 and Notch-1 gene activation, which is implicated in the self-renewal of Hematopoietic stem cells (HSC)s [151].	➢ Colon carcinoma and blood diseases ➢ β-catenin accumulation has been associated with breast or brain cancer, melanoma, and myeloid leukemia [162]. ➢ β-catenin mutations observed in hepato-cellular, endometrial, and prostate carcinomas [162].
**PTEN**	➢ Hematopoietic stem cells and neural stem cells maintenance	➢ Loss of expression of Pten in mice results in aberrant self-renewal of HSCs and eventually leukemia [163]. ➢ Loss of Pten eventually leads to myelo-proliferative disease and the emergence of a transplantable leukemia. ➢ Mutations and/or loss of heterogeneity of Pten can cause glioblastoma, prostate, and endometrial carcinoma [164].
**Sonic hedgehog (Shh)**	➢ Bmi-1 activation in the brain [165]. ➢ The Shh signaling pathway is essential for the embryonic development of hair follicles and sebaceous glands [166]. ➢ Shh signaling pathway is implicated in postnatal and adult brain development [167].	➢ Shh activation leads to both skin and brain carcinogenesis [168]. ➢ Shh mutation leads toGorlin’s syndrome [168].
**Hox family**	➢ HSCs self-renewal [169].	➢ Overexpressed in T-ALL with chromosome translocations [159]. ➢ Hoxb 3, 8, and 10 are associated with leukemo-genesis in mice [169]. ➢ HoxA9 is over-expressed in AML patients [169].

**Table 2:** The differences between cancer stem and normal stem cells.

	Cancer stem cells	Normal stem cells
**Surface** **markers**	AML (CD123^+^/CD117^–^), Prostate (CD133^+^/^–^), Breast (CD44^+^/CD24^–^)	Absent
**Self-renewal** **capacity**	Extensive and indefinite	Limited
**Nature**	Tumorigenic	Organogenic
**Karyotype**	Abnormal	Normal
**Tumor** **suppressor** **genes**	Present (Interferon factor-1, Death associated protein kinase-1)	Absent

For a better understanding of CSCs biology, we must know the unique properties of normal stem cells. Normal stem cells are defined by an extensive capacity for self-renewal and by their ability to undergo a broad range of differentiation. ESCs are omnipotent and have limitless replicative life span, which is ascribed to their telomerase expression [31]. Much effort has been devoted to the identification and characterization of CSCs [32,33,34,35]. For isolation of CSCs, fractionation of tumor cells using cell-surface markers characteristic of stem cells can be used. The CD133 cell-surface marker was used to purify putative CSCs in several tumor types, with the exception of breast [36], prostate [37] and head and neck carcinomas [31] where CD44 was utilized instead. CD133 (prominin-1) was discovered as a marker of normal hematopoietic stem cells and later was found to mark stem/progenitor cells from a wide variety of tissues [38]. CSCs have been isolated from cancers of the breast, brain, blood (leukemia), skin (melanoma), head and neck, thyroid, cervix, lung, gastrointestinal tract, reproductive tract, and retina [39].

### 4.1. Breast CSCs

As epithelial CSCs, we focus in this review on breast CSCs. Despite recent breakthroughs in mouse mammary stem cells and lineage determination in mammary glands, little has been determined in human mammary stem cells. Breast cancer is one of the major causes of cancer-related deaths in women; in the USA alone, more than 40,000 breast cancer fatalities occur annually. The origin of breast CSCs is from mammary multipotent stem cells as a result of genetic defects caused by deleterious agents that affect pathways controlling self-renewal and differentiation [40]. Importantly, breast CSCs have been shown to express higher levels of oxidative stress-responsive genes, which could confer part of their ability to resist anticancer therapy, compared to non-CSCs [41]. Several studies indicate that breast cancer is caused by CSCs, and the cure of breast cancer requires eradication of breast CSCs [42,43]. Basically, the adult human mammary gland is composed of a series of branched ducts and lobular-alveolar units embedded in fatty tissue and is composed of three forms of the basal layer of ducts and alveoli; (a) Myo-epithelial cells which express the a form of smooth muscle actins (SMA), common acute lymphoblastic leukemia antigen (CALLA, also known as CD10 and CK14 (b) ductal epithelial cells which express MUC-1, CK8, CK18 and CK19; and (c) Alveolar epithelial cells [44]. 

There are several signaling pathways controlling the self-renewal ability of human and mouse normal and malignant mammary stem cells such as Notch [45], Hedgehog [46], Wnt/b-catenin [47], epidermal growth factor (EGF)-like/EGF receptor (EGFR)/Neu [41], leukemia inhibitory factor (LIF) [48], TGF-β [49], integrins [50], telomerase [51], SDF-1/CXCR4 [52], the insulin-like growth factor-1 (IGF-1) system [53], and ER/PR [53]. The identification of markers of a breast CSC really began the current excitement [21], and the identification of CSCs in various cancer types using candidate surface markers is an area of active research. Primary human breast cancer cells are immuno-phenotypically heterogeneous and CD44^+^ subpopulations are tumorigenic in NOD/SCID mice bearing estrogen pellets [21]. Breast CSCs, or tumor-initiating cells, can be isolated by the immuno-sorting of breast cancer cells that express the hyaluronian receptor CD44, a gene that is overexpressed in basal-like tumors [54] and lack the expression of CD24, an endogenous inhibitor of the chemokine receptor CXCR [21,55]. CD44-positive cells isolated from ductal breast carcinoma and from normal mammary glands were found to express low levels of ER alpha and high levels of CK5 [56]. For better understanding of putative breast CSCs at the molecular level, Shipitsin *et al.* carried out SAGE (serial analysis of gene expression) profiling of CD24^−/low^/CD44^+^ and CD24^+^/CD44^+/−^cell populations from normal and neoplastic human breast tissue. The identification of new markers was mainly based on the CD44^+^/CD24^−^ specific criteria to isolate breast CSCs. By using gene expression profiling of CD44 positive cells from breast carcinoma-derived pleural effusions, Shipitsin *et al.* identified a CD44 positive cell-specific gene, PROCR. PROCR encodes a cell surface receptor and its expression is specific to CD44 positive epithelial cells [56]. CD133 is a known marker of CSCs in several organs including brain, blood, liver, and prostate [57,58,59]. Interestingly, they found that the CD133^+^ stem cell-like population did not overlap with the CD44^+^/CD24^−^ population and that both populations had a similar capacity for self-renewal and could reconstitute cell fractions found in the respective parental cells [60]. This finding suggests that there might be different kinds of breast cancer stem cell subpopulations that express surface markers other than CD44. NF-κB–regulated genes play a fundamental role in mammary gland morphogenesis, therefore, pointing out a primary role in the regulation of stem cells [61,62]. Recently, it was observed that the inhibition of NF-κB activity halts mammosphere (MS) formation from mouse and human mammary glands [63]. Overexpression of NF-κB–regulated genes in CD44 positive breast CSCs was found, and this finding is similar to what occurs to hemopoietic stem cells [64]. The upregulation of NF-κB–regulated targets in CD44 positive breast CSCs may be functionally linked to the overexpression of hypoxia-induced factor 1-alpha (HIF-1α) in such cells, in the absence of a hypoxic environment [56]. The expression profiles of stem-like cells from normal and neoplastic breast tissue were highly similar, and both expressed numerous stem cell markers, whereas both normal and breast cancer CD24^+^/CD44^+/−^ cells had features of luminal differentiation. 

### 4.2. Prostate CSCs

The prostate is a hormonally regulated male secretary organ composed of a multitude of cells, some of which possess renewal properties [65,66]. Recently, several laboratories have developed interest in the isolation and characterization of candidate prostate CSCs from both mouse and human prostates. Normal human prostatic basal cells express the cell adhesion molecule CD44 [67]. Recently, CD44 isoforms, or splice variants, have been evidenced to be a marker of CSCs in a variety of tissues, including the breast and prostate [68,69].

### 4.3. Neuronal CSCs

Isolation of central nervous system (CNS) CSCs has been carried out by means of antigenic markers and by exploiting *in vitro* culture conditions developed for normal neural stem cells. CNS cells grown on nonadherent surfaces, as was first detected in 1992 [70,71], give rise to neurospheres (balls of cells) that have the capacity for self-renewal and can give rise to all of the principal cell types of the brain (*i.e*., oligodendrocytes, neurons, and astrocytes).

### 4.4. ROS and CSCs

ROS production from cells occurs via multiple mechanisms. A major source of ROS is produced in the mitochondria. In comparison to normal cells, malignant cells seem to function with higher levels of endogenous oxidative stress *in vitro* and *in vivo* [72,73]. High levels of oxidative stress have been observed in various types of cancer cells. For instance, leukemia cells freshly isolated from blood samples of patients with chronic lymphocytic leukemia showed increased ROS production in comparison to normal lymphocytes [74,75]. Importantly, the levels of ROS-scavenging enzymes such as superoxide dismutases (SOD), glutathione peroxidase and peroxiredoxin have been shown to be significantly altered in cancer cells [76] and in primary cancer tissues [77,78]. Interestingly, the alterations in ROS-scavenging enzymes such as GSH also have a significant effect on the metabolism of alkylating agents [79,80]. Accordingly, there is an aberrant regulation of redox homeostasis and stress adaptation in cancer cells. In order to overcome the drug resistance associated with redox adaptation, it is important to design a strategy that exploits the redox difference between normal cells and cancer cells, and that disables the redox adaptation mechanism in cancer cells. Therefore, targeting the key redox regulatory mechanisms that control both the level of ROS and the function of redox sensitive survival proteins is considered as one of such strategies. The thiol-based antioxidants GSH, thioredoxin and peroxiredoxin can be considered potential candidates for such a redox intervention strategy. As recent studies proved, rapid depletion of GSH using the natural compound PEITC can not only kill *Ras*-transformed ovarian cells and primary leukemia cells from patients, but can also eliminate the drug-resistant cell population [81,81]. Although the redox status of CSCs is not yet clear, it is possible that cancer and normal stem cells could share some common features while exhibiting malignant cell characteristics in redox regulation [10]. It is very interesting to mention that recent studies proved that normal hemopoietic stem cells and normal mammary epithelial stem cells maintain ROS at lower levels than their mature progeny to prevent cellular differentiation and maintain long-term self-renewal [82,83,84]. In comparison to normal cells, cancer cells have higher levels of ROS, which seems to be essential for malignant initiation and progression [85]. Interestingly; there are subsets of CSCs in human and mouse breast tumors containing lower ROS levels than the corresponding non-tumorigenic cells [84]. This low level of ROS seems to be associated with high expression of ROS-scavenging molecules, which may contribute to tumor radio-resistance [84] Moreover, the unchecked ROS accumulation is thought to play a part in the conversion from normal hemopoietic stem cells to leukemic cells [96,99]. Collectively, in regard to the biological properties of CSCs, this unique cell subpopulation might have a high antioxidant capacity to keep cellular ROS at a moderate level and maintain both stemness and cancer-forming capabilities. Moreover, the highly upregulated antioxidant mechanisms might contribute to CSCs survival and drug resistance.

## 5. ROS and Apoptosis

Cells respond to stress in various ways, ranging from activation of pathways that promote survival to eliciting programmed cell death that eliminates damaged cells. The initial response of cells to stressful stimuli is geared toward helping the cells to defend against and recover from the insults. Cells activate death signaling pathways if the noxious stimulus is unresolved. Cell death has many forms and shapes. The research of cell death includes not only the study of programmed forms of cell death (both apoptosis and autophagic cell death), necrosis and other modes of cellular demise, but also the essential roles of these phenomena in various physiological and pathological processes such as development, aging, and disease. In the last two decades, the cell death field has attracted much attention, mainly because of its role in development and cancer [88]. Apoptosis, or programmed cell death (PCD), is a naturally occurring cell death process, which is crucial for the normal development and homeostasis of all multicellular organisms [89]. Apoptotic cell death may be triggered through the extrinsic (receptor-mediated) or the intrinsic (mitochondria-mediated) pathway. The intrinsic pathway can be triggered by many stimuli including ROS. Mitochondria are the major site of ROS production and accumulation of ROS may lead to the initiation of apoptosis [90]. Many cytotoxic agents induce ROS, including peroxide and O_2_*•^−^*, which are involved in the induction of apoptotic cell death [91]. H_2_O_2_ can cause the release of cytochrome *c* from mitochondria into the cytosol. Moreover, H_2_O_2_ may also activate nuclear transcription factors, such as NF-κB, AP-1, and p53 [92], which may lead to upregulation of death proteins or production of inhibitors of survival proteins. Several studies imply that inhibition of apoptosis by Bcl-2 is associated with protection against ROS [93]. High oxidative stress level kills cells either by necrosis or by apoptosis [94,95]. In various apoptosis models, changes in the redox status of the cells to a more oxidizing environment occurs prior to activation of the final phase of caspase activation [95,96]. This case is further supported by the ability of various antioxidants such as *N*-acetylcysteine (NAC) to block apoptosis in a similar way that caspase inhibitors do [97]. In addition, the antioxidant properties of Bcl-2, a potent inhibitor of apoptosis, further confirm this view [89,99]. Under normal conditions, aerobic cells are endowed with extensive antioxidant defense mechanisms to counteract the damaging effects of ROS [100,101]. When prooxidants overwhelm antioxidant defense mechanisms, oxidative stress occurs. Interestingly, apoptosis may serve as a fail-safe device to prevent cells from proliferating uncontrollably in the face of a persistent oxidative stress [102]. Interestingly, current chemotherapeutic agents such as anthracycline-derivatives, which are frequently used as chemotherapeutics in the treatment of various types of cancers, target some of these apoptotic pathways. For example, adriamycin is known to chelate iron and generate ROS that result in apoptosis of cancer cells [103]. Another example of a chemotherapeutic agent that generates ROS for cancer treatment is artesunate (ART), which induces apoptosis in leukemic T cells mainly through the mitochondrial pathway via ROS generation [104].

## 6. ROS and Autophagy

Autophagy (self-eating), an evolutionarily conserved multistep process, is characterized by the vesicular sequestration and degradation of long-lived cytoplasmic proteins and organelles, for example, mitochondria [105]. It is classified as Type II programmed cell death [106]. It is characterized by double-membraned vacuoles, autophagosomes, and requires the two ubiquitin-like conjugation systems (Atg12 and Atg8 ligation systems) and activation of class III phosphatidylinositol-3-kinase [107,108]. Deregulation of the autophagy process may lead to cancer, neurodegenerative disorders, and cardiovascular diseases [109]. Of note, autophagy is up-regulated during stress or any physiological change. By breaking-down longer-lived proteins and organelles and recycling the products into protein-synthesis and energy-production pathways, the process allows cells to be temporarily self-sustaining during periods when nutrients are restricted [110,111]. DNA damage-activation response is a hallmark of oxidative stress caused by ROS. Protein re-folding in the endoplasmic reticulum (ER) by protein disulfide isomerases can elevate oxidative stress through redox reactions involving free radicals [112], and mitochondrial stress and damage can also be a source of ROS in autophagy-deficient cells [113]. Interestingly, ROS play a pivotal role in the induction of cadmium (Cd)-induced autophagy, as Cd is able to induce autophagic cell death through a calcium-extracellular signal-regulated kinase (ERK) pathway [114]. A recent study demonstrated that, in MES-13 mesangial cells, cadmium-induced autophagy was mediated through the ROS-glycogen synthase kinase-3β (GSK-3β) signaling pathway. In addition, both Cd-induced ROS bursts and autophagy were reduced by ROS scavenger, N-acetylcysteine (NAC) and vitamin E [115]. Mitochondria may play a central role in the mechanism of autophagy-induced cell death [116], and autophagy often occurs when the mitochondria fail to maintain ATP levels [117] or when the mitochondria are damaged [116]. ROS are often generated following inhibition of the mitochondrial electron transport chain (mETC) [118,119,120]. It is estimated that 2% of oxygen is converted to ROS by mETC [130]. Accordingly, it is very interesting to mention that selective prolonged activation of autophagy, such as treatment with mETC inhibitors of complex I (rotenone) and II (TTFA) in cancer cells, could be a viable strategy to treat cancers resistant to apoptosis. Emerging proof shows that the imbalance in the homeostasis of the oxidative condition of cells through the caspase inhibition or starvation leads to autophagy induction [121,122]. Moreover, induction of autophagy by starvation occurs through inactivation of HsAtg4A, an oxidant-sensitive cysteine protease, by ROS, resulting in accumulation of Atg8-PE [121]. All the forementioned examples suggest that ROS may be one of the major mediators in the regulation of autophagy. 

## 7. Signaling Pathways, Transcription Factors, and Their Roles in Oxidative Stress

**Figure 2:**
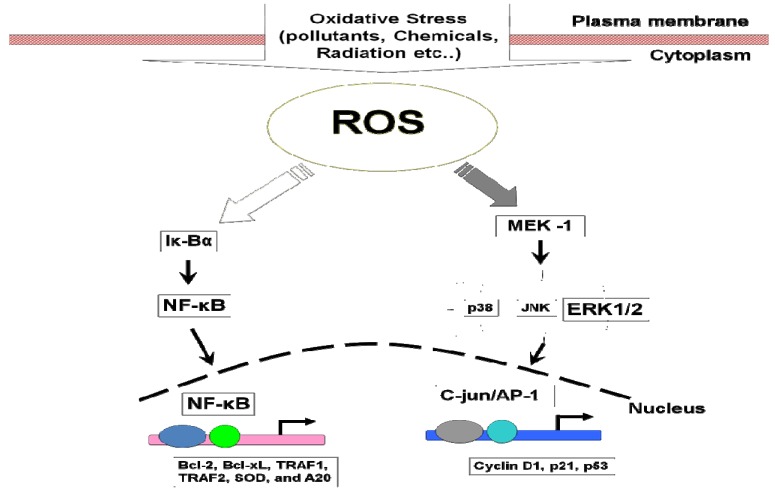
ROS mediated activation of cell signaling pathways.

ROS can stimulate cellular proliferation and activate survival pathways via several signaling mechanisms. ROS-induced DNA damage has been widely accepted as a major cause of cancer [123]. There are several signaling pathways and transcription factors controlling oxidative stress in cancer development, such as those shown in Figure 2 below.

### 7.1. Mitogen-Activated Protein Kinases (MAPKs)

The Mitogen-activated Protein Kinases (MAPKs) are a family of serine/threonine kinases involved in various cellular processes such as, energy metabolism, regulation of gene expression, and programmed cell death [124,125]. The implication of MAPK pathways in both cell proliferation and death is emphasized by the observation that deregulation of these kinase cascades can result in cell transformation and cancer [126]. Oxidants have been shown to be able to trigger the activation of multiple signaling pathways, including the phosphorylation cascades of MAPKs. There are three structurally related, but functionally distinct MAPKs, which are extracellular signal-regulated Kinase (ERK), c-Jun N-terminal kinase (JNK), and p38 MAPK [127]. ERKs can be stimulated by mitogens, while JNK and p38 MAPK can be activated by heat shock proteins and inflammatory cytokines [128]. 

#### 7.1.1. MAPKs and Cancer

Relatively high level oxidative stress activates the stress signal cascades of JNK, NF-κB and other pathways. On the other hand, low levels of oxidative stress were shown to selectively activate the p38 MAPK-related cascade leading to abnormal cell cycle progression [129]. ROS trigger signaling cascades, which lead to the activation and phosphorylation of MAPKs, including ERK. As a consequence, transcription factors including NF-κB and AP-1 are activated, which may lead to the induction of early response genes such as c-jun and c-fos, which are involved in inflammatory influx, inhibition of apoptosis, cell proliferation, transformation, differentiation, and other changes [130]. Activation of the ERK, JNK, and p38 MAPK subfamilies has been observed in response to changes in the cellular redox balance. The balance between ERK and JNK activation is a key determinant for cell survival, as both a decrease in ERK and an increase in JNK are essential for the induction of apoptosis [131]. There is strong proof that suggests that many protein kinases and their corresponding transcriptional regulatory factors are activated under oxidative stress conditions [129]. The ERK pathway primarily controls the processes of proliferation and survival, while the JNK pathway can promote either proliferation or apoptosis [132]. Activation of both ERK and JNK pathways can lead to increased proliferation and survival, although loss of JNK in some instances may also promote tumorigenesis [86]. On the other hand, the p38 MAPK pathway is activated upon cellular stress and often engages pathways that can block proliferation or promote apoptosis [133]. Interestingly, p38 MAPK selectively functions as a sensor of oxidative stress during the initiation of tumorigenesis [134,135]. 

#### 7.1.2. MAPKs and Stem Cells

Inhibition of p38 MAPK appears to maintain pluripotency by blocking the pro-differentiation effects of p38 MAPK [140], ROS activates the p38/MAPK pathway causing quiescent HSCs to cycle more frequently and eventually become exhausted [141]. The mutant HSCs show increased phosphorylation of p38 MAPK, a heightened sensitivity to cell cycle-specific myelotoxic injury, and lose self-renewal capacity during aging. Several cellular growth- and proliferation-related signal transduction pathways are activated by ROS signaling. Among of these are MAPK and the redox sensitive kinases [142].The oxidative stress microenvironment plays an important role in the clonal evolution of tumor progression by permitting/potentiating genetic instability, epigenetic modulation of gene expression, and the activation of growth and survival-related signal transduction pathways [143].

#### 7.1.3. MAPKs and CSCs

A recent study demonstrated that CSCs can be derived from human mammary epithelial cells following the activation of the Ras-MAPK pathway [136]. The acquisition of these stem and tumorigenic characters is driven by Epithelial-Mesenchymal Transition (EMT) induction. MAPK/ERK1, 2 and vascular endothelial growth factor 1 (VEGF/Flt1) autocrine pathways may play significant roles in drug-induced expansion of bone marrow side-population (SP) cells (G0 phase) [137,138]. siRNA inhibition of Flt1 reduced nanog [139] and Oct-4 expression [138], suggesting that stress-induced activation of the VEGF/Flt1 and MAPK/ERK1,2 autocrine loop may play an important role in the expansion of the CSCs fraction.

### 7.2. NF-κB

The nuclear factor kappa B (NF-κB) represents a typical example of a transcription factor whose activity can be significantly changed via redox modulation. NF-κB plays an important role in the regulation of many genes involved in immune, inflammatory, and antiapoptotic responses. Therefore, this molecule has a crucial role in controlling cell survival in response to oxidative insults.

#### 7.2.1. NF-κB and Cancer

NF-κB activation has been associated with the carcinogenesis process, because of its roles in cell growth and differentiation and its role in inflammation. Moreover, NF-κB regulates several genes implicated in cell transformation, proliferation, and angiogenesis [9]. Inside the cells, NF-κB is normally bound to the inhibitory protein Iκ-Bα in the cytoplasm. ROS activate NF-κB by rapid phosphorylation, ubiquitination, and subsequent proteasomal degradation of the inhibitory protein Iκ-Bα. This is followed by the translocation of NF-κB to the nucleus, where it activates gene transcription (Figure 2) [144]. Continuous production and high levels of ROS lead to activation of NF-κB, which through the activation of various pro-inflammatory cytokines produces chronic inflammation that subsequently ends in tumor development [144]. Interestingly, carcinogenic promoters such as UV radiation, asbestos, alcohol, and phorbol esters are among the exogenous sources of ROS that activate NF-κB [9]. Consequently, this leads to activation of the expression of several genes regulated by NF-κB such as, bcl-2, bcl-x_L_, TRAF1, TRAF2, SOD, and A20, which promote cell survival through inhibition of apoptotic pathways (Figure 1). NF-κB is a transcription factor, which has an essential role in the expression of many genes whose products can suppress tumor cell death; stimulate tumor cell cycle progression; enhance epithelial-to-mesenchymal transition, which has an important role in tumor invasiveness; and provide newly emerging tumors with an inflammatory microenvironment that supports their progression, invasion of surrounding tissues, angiogenesis, and metastasis [145,146]. NF-κB is a transcription factor that can induce the expression of IL-6, a cytokine that plays an essential role in the immune response and inflammation [147]. Of note, tumor cells from breast, colon, blood neoplasms, pancreas, and squamous cell carcinoma cell lines have all been reported to constitutively express activated NF-κB [148].

#### 7.2.2. NF-κB and Stem Cells

NF-κB is a transcriptional regulator involved in many biological processes including proliferation, survival, and differentiation. Recently, it has been shown that members of the NF-κB family are widely expressed by neurons, glia, and neural stem cells [149]. NF-κB, an inducible dimeric transcription factor that belongs to the Rel family of transcription factors, is a major mediator of the cellular response to a variety of extracellular stimuli and is involved in diverse biological processes including embryo development, hematopoiesis, and immune regulation, as well as neuronal functions via the induction of certain growth and transcription factors [150,151]. There are five different Rel/NF-κB proteins expressed in mammals: p65 (RelA), p50 (NF-κB1), p52 (NF-κB2), c-Rel (Rel), and RelB [152]. In mouse ES cells, there is a lower expression of NF-κB p65 protein in comparison to mouse embryonic fibroblast cells. These NF-κB proteins form homo- or heterodimers and are bound in the cytoplasm by the inhibitor of κB proteins (IκB) [153]. A recent study reported that expression and activity of the transcription factor NF-κB was enhanced during differentiation of human ES cells [154].

#### 7.2.3. NF-κB and CSCs

In the case of CSCs, oxidative stress activates NF-κB signaling. Such activation allows NF-κB dimers to translocate to the nucleus and to activate transcription of target genes. Basically, the NF-κB pathway regulates genes involved in key cellular processes such as proliferation, stress response, innate immunity, and inflammation [63]. NF-κB signaling is necessary to maintain pluripotency in human ESCs. These findings might support the hypothesis that stem cells might undergo transformation into CSCs under prolonged oxidative stress, probably due to molecular modifications such as hyperoxia-induced NF-κB [123]. Therefore, the ROS status related to CSCs and their ability to self-renew and escape death signals need to be fully elucidated.

## 8. Conclusions

Several recent scientific reviews and studies have enthusiastically described the relationship between the increase in cellular reactive oxygen radicals and the pathogenesis of several chronic diseases, including cancer. There are two sources of cellular oxidants (reactive oxygen and nitrogen species) that can be generated from endogenous (normal physiological processes) as well as exogenous sources (xenobiotic interaction). When the antioxidant control mechanisms are overrun, the cellular redox potential shifts toward oxidative stress. As a consequence, the potential for damage to cellular nucleic acids, lipids, or proteins increases. Importantly, oxidative nuclear DNA damage has an important role in neoplasia. Cancer cells exhibit increased ROS generation that may promote cell proliferation. This might be coupled to redox adaptation to promote cell survival and drug resistance. These highlight the crucial role of ROS stress in tumor development and drug resistance. Accordingly, there is a growing scientific need for the identification of the key molecular mechanisms that determine the redox balance in cancer stem cells, which might provide a possibility to terminate the survival mechanisms in these cells and enable the elimination of cancer from its root. Moreover, formation of mitochondrial DNA damage, mutation, and alteration of the mitochondrial genomic function also seem to be implicated in the process of carcinogenesis. It is worth noting that ROS and cellular redox status mediate cell signaling pathways that are implicated in cell growth regulatory pathways and, in consequence, carcinogenesis. Importantly, the role of ROS in the regulation of cell growth is very complicated, as it is cell specific and depends upon the form of the oxidant as well as the concentration of the particular ROS. Interestingly, gene expression modification by ROS has direct effects on cell proliferation and apoptosis through the activation of transcription factors including MAPK/AP-1 and NF-κB pathways. In this review, we summarize the current knowledge on the link between oxidative stress, different signaling pathways, and carcinogenesis, by focusing in particular on the relations of both the MAPK family of signaling pathways and the transcription factor NF-κB to oxidative stress and the carcinogenesis process. Both the MAPK pathways and the transcription factor NF-κB may have essential roles in the redox status and the development of cancer stem cells. Most importantly, we describe the relation between ROS and both apoptosis and autophagy, and in turn, to tumorigenesis. There is increasing evidence supporting that oxidative stress and both apoptosis and autophagy are closely linked physiological phenomena. Autophagy, a cellular self-catabolic process, can be cytotoxic and under certain settings substitute for apoptosis in induction of cell death. In addition, loss of autophagy is related to tumorigenesis. The relation of autophagy to tumorigenesis is complex and depends on the genetic composition of cells as well as on the extra-cellular stresses which a cell is exposed to. The relationship between oxidative stress and both apoptosis and autophagy may have a crucial role in cancer stem cell development as well as therapy. In order to validate and confirm all of these aforementioned notions, more in-depth further studies and investigations are needed. This perhaps will provide insights for the development of novel therapeutic strategies.

## References

[1] Vishal R.T., Sharma S., Mahajan A., Bardi G.H. (2005). Oxidative Stress: A Novel Strategy in Cancer Treatment. JK Sci..

[2] Chandra J., Samali A., Orrenius S. (2000). Triggering and modulation of apoptosis by oxidative stress. Free Rad. Med. Biol..

[3] Poli G., Biasi F., Chiarpotto E. (2004). Oxidative stress and cell signaling. Curr. Med. Chem..

[4] Pillai C.K., Pillai K.S. (2002). Antioxidants in health. Int. J. Physiol. Pharmacol..

[5] Sohal R.S. (1996). Oxidative stress, caloric restriction, and aging. Science.

[6] Valko M., Rhodes C.J., Moncol J., Izakovic M., Mazur M. (2006). Free radicals, metals and antioxidants in oxidative stress-induced cancer. Chem. Biol. Interact..

[7] Inoue M., Nishikawa M., Park A.M., Kira Y., Imada I., Utsumi K. (2003). Mitochondrial generation of reactive oxygen species and its role in aerobic life. Curr. Med. Chem..

[8] Parke D.V. (1996). Chemical toxicity and reactive oxygen species. Int. J. Occup. Med. Environ. Health.

[9] Waris G., Ahsan H. (2006). Reactive oxygen species: Role in the development of cancer and various chronic conditions. J. Carcinog..

[10] Ogasawara M.A., Zhang H. (2009). Redox regulation and its emerging roles in stem cells and stem-like cancer cells. Antioxid. Redox Signal..

[11] Nesti C., Pasquali L., Mancuso M., Siciliano G., Vinagolu K.R., Mohan C.V. (2009). The Role of Mitochondria in StemCell Biology. Stem Cell Biology and Regenerative Medicine Regulatory Networks in Stem Cells.

[12] Thomson J.A., Odorico J.S. (2000). Human embryonic stem cell and embryonic germ cell lines. Trends Biotechnol..

[13] Serakinci N, Keith N.W. (2006). Therapeutic potential of adult stem cells. Eur. J. Cancer.

[14] Jacks T., Weinberg R.A. (1996). Cell-cycle control and its watchman. Nature.

[15] Sugimura T. (1998). A new concept of co-mutagenicity from a phenomenon forgotten for the past two decades: Is it more important than previously expected?. Environ. Health Perspect..

[16] Visvader J.E., Lindeman G.J. (2008). Cancer stem cells in solid tumours: accumulating evidence and unresolved questions. Nat. Rev. Cancer.

[17] Eyler C.E, Rich J.N. (2008). Survival of the fittest: cancer stem cells in therapeutic resistance and angiogenesis. J. Clin. Oncol..

[18] Li X., Lewi M.T., Huang J., Gutierrez C., Osborne C.K., Wu M., Hilsenbeck S.G., Pavlick A., Xiaomei Zhang X., Chamness G.C., Wong H., Rosen J., Chang J.C. (2008). Intrinsic resistance of tumorigenic breast cancer cells to chemotherapy. J. Natl. Cancer Inst..

[19] Bao S., Wu Q., McLendon R.E., Hao Y., Shi Q., Hjelmeland A.B., Dewhirst M.W., Bigner D.D., Rich J.N. (2006). Glioma stem cells promote radioresistance by preferential activation of the DNA damage response. Nature.

[20] Dalerba P., Cho R.W., Clarke M.F. (2007). Cancer stem cells: models and concepts. Annu. Rev. Med..

[21] Wicha M.S., Liu S., Dontu G. (2006). Cancer stem cells: an old idea—a paradigm shifts. Cancer Res..

[22] Al-Hajj M., Wicha M.S., Benito-Hernandez A., Morrison S.J., Clarke M.F. (2003). Prospective identification of tumorigenic breast cancer cells. Proc. Natl. Acad. Sci. USA.

[23] Collins A.T., Berry P.A., Hyde C., Michael J., Stower M.J., Maitland N.J. (2005). Prospective identification of tumorigenic prostate cancer stem cells. Cancer Res..

[24] Soltysova A., Altanerova V., Altaner C. (2005). Cancer stem cells. Neoplasma.

[25] Jones R.J., Matsui W.H., Smith B.D. (2004). Cancer stem cells: are we missing the target?. J. Natl. Cancer Inst..

[26] Dontu G., Jackson K.W., McNicholas E., Kawamura M.J., Abdallah W.M., Wicha M.S. (2004). Role of Notch signaling in cell-fate determination of human mammary stem/progenitor cells. BreastCancer Res..

[27] Lowe S.W., Sherr C.J. (2003). Tumor suppression by Ink4a-Arf: Progress and puzzles. Curr. Opin. Genet. Dev..

[28] Hatsell S., Frost A.R. (2007). Hedgehog signaling in mammary gland development and breast cancer. J. Mammary Gland Biol. Neoplasia.

[29] Lindvall C., Bu W., Williams B.O. (2007). Wnt signaling, stem cells, and the cellular origin of breast cancer. Stem Cell Rev..

[30] Zhou J., Wulfkuhle J., Zhang H., Gu P., Yang Y., Deng J., Margolick J.B., Liotta L.A., Petricoin  E., Zhang Y.  (2007). Activation of the PTEN/mTOR/STAT3 pathway in breast cancer stem-like cells is required for viability and maintenance. Proc. Natl. Acad. Sci. USA.

[31] Prince M.E., Sivanandan R., Kaczorowski A., Wolf G.T., Kaplan M.J., Dalerba P., Weissman I.L., Clarke M.F., Ailles L.E. (2007). Identification of a subpopulation of cells with cancer stem cell properties in head and neck squamous cell carcinoma. Proc. Natl. Acad. Sci. USA.

[32] Shmelkov S.V., St Clair R., Lyden D., Rafii S. (2005). AC133/CD133/Prominin-1. Int. J. Biochem. Cell Biol..

[33] Mimeault M., Batra S.K. (2007). Recent advances in cancer stem/progenitor cell research: therapeutic implications for overcoming resistance to the most aggressive cancers. J. Cell. Mol. Med..

[34] Kai K., Arima Y., Kamiya T., Saya H. (2009). Breast cancer stem cells. Breast Cancer.

[35] Zhang M., Rosen J.M. (2006). Stem cells in the etiology and treatment of cancer. Curr. Opin. Genet. Dev..

[36] Sun W., Kang K.S., Morita I., Trosko J.E., Chang C.C. (1999). High susceptibility of a human breast epithelial cell type with stem cell characteristics to telomerase activation and immortalization. Cancer Res..

[37] Benny K., Abraham B.K., Fritz P., McClellan M., Hauptvogel P., Athelogou M., Brauch H. (2005). Prevalence of CD44^+^/CD24^−/low^ cells in breast cancer may not be associated with clinical outcome but may favor distant metastasis. Clin. Cancer Res..

[38] Kritikou E.A., Sharkey A., Abell K., Came P.J., Anderson E., Clarkson R.W.E., Watson C.J. (2003). A dual, non-redundant, role for LIF as a regulator of development and STAT3-mediated cell death in mammary gland. Development.

[39] Boulanger C.A., Wagner K.U., Smith G.H. (2005). Parity-induced mouse mammary epithelial cells are pluripotent, self-renewing and sensitive to TGFb1 expression. Oncogene.

[40] Shipitsin M., Campbell L.L., Argani P., Weremowicz S., Bloushtain-Qimron N., Yao J., Nikolskaya T., Serebryiskaya T., Beroukhim R., Hu M., Halushka M.K., Saraswati S., Parker L.M., Anderson K.S., Lyndsay N., Harris L.N., Garber J.E., Richardson A.L., Schnitt S.J., Nikolsky Y., Gelman R.S., Polyak K. (2007). Molecular definition of breast tumor heterogeneity. Cancer Cell.

[41] Azad N., Rojanasakul Y., Vallyathan V. (2008). Inflammation and lung cancer: Roles of reactive oxygen/nitrogen species. J. Toxicol. Environ. Health B.

[42] Vercauteren S.M., Sutherland H.J. (2001). CD133 (AC133) expression on AML cells and progenitors. Cytotherapy.

[43] Kurata S. (2000). Selective activation of p38 MAPK cascade and mitotic arrest caused by low level oxidative stress. J. Biol. Chem..

[44] Marshall H.E., Hess D.T., Stamler J.S. (2004). S-Nitrosylation: Physiological regulation of NF-kappaB. Proc. Natl. Acad. Sci. USA.

[45] Xia Z., Dickens M., Raingeaud J., Davis R.J., Greenberg M.E. (1995). Opposing effects of ERK and JNK-p38MAPkinases on apoptosis. Science.

[46] Demicco E.G., Kavanagh K.T., Romieu-Mourez R., Xiaobo Wang X., Shin S.R., Esther Landesman-Bollag E., Seldin D.C., Sonenshein G.E. (2005). RelB/p52 NF-kappaB complexes rescue an early delay in mammary gland development in transgenic mice with targeted superrepressor IkappaB-alpha expression and promote carcinogenesis of the mammary gland. Mol. Cell Biol..

[47] Cao Y., Luo J.L., Karin M. (2007). IkappaB kinase alpha kinase activity is required for self-renewal of ErbB2/Her2-transformed mammary tumor-initiating cells. Proc. Natl. Acad. Sci. USA.

[48] Kasper M., Jaks V., Marie Fiaschi M., Rune Toftgård R. (2009). Hedgehog signalling in breast cancer. Carcinogenesis.

[49] Lam J.S., Reiter R.E. (2006). Stem cells in prostate and prostate cancer development. Urol. Oncol..

[50] Tang D.G., Patrawala L., Calhoun T., Bhatia B., Schneider-Broussard R., Choy G., Jeter C. (2007). Prostate cancer stem/progenitor cells: identification, characterization and implications. Mol. Carcinogen.

[51] Liu A.Y., True L.D., LaTray L., Nelson P.S., Ellis W.J., Vessella R.L., Lange P.H., Hood L., Van Den Engh G. (1997). Cell-cell interaction in prostate gene regulation and cytodifferentiation. Proc. Natl. Acad. Sci. USA.

[52] Patrawala L., Tang D.G., Bertolotti R., Ozawka K. (2008). CD44 as a functional cancer stem cell marker and therapeutic target. Progress in Gene Therapy: Autologous and Cancer Stem Cell Gene Therapy.

[53] Ricci-Vitiani L., Lombardi D.G., Pilozzi E., Biffoni M., Todaro M., Peschle C., De Maria R. (2007). Identification and expansion of human colon-cancer-initiating cells. Nature.

[54] Reynolds B.A., Weiss S. (1992). Generation of neurons and astrocytes from isolated cells of the adult mammalian central nervous system. Science.

[55] Honeth G., Bendahl P., Ringnér M., Saal L.H., Gruvberger-Saal S.K., Lövgren K., Grabau D., Fernö M., Borg A., Hegardt C. (2008). The CD44^+^/CD24^− ^phenotype is enriched in basal-like breast tumors. Breast Cancer Res..

[56] Bertucci F., Finetti P., Cervera N., Charafe-Jauffret E., Mamessier E., Adélaïde J., Debono S., Houvenaeghel G., Maraninchi D., Viens P., Charpin C., Jacquemier J., Birnbaum D. (2006). Gene expression profiling of breast cell lines identifies potential new basal markers. Oncogene.

[57] Mark A., La Barge M.A., Bissell M.J. (2008). Is CD133 a marker of metastatic colon cancer stem cells?. J. Clin. Invest..

[58] Singh S.K., Clarke I.D., Hide T., Dirks P.B. (2004). Cancer stem cells in nervous system tumors. Oncogene.

[59] Vercauteren S.M., Sutherland H.J. (2001). CD133 (AC133) expression on AML cells and progenitors. Cytotherapy.

[60] Yin S., Li J., Hu C., Chen X., Yao M., Yan M., Jiang G., Ge C., Xie H., Wan D., Yang S., Zheng S., Gu J. (2007). CD133 positive hepatocellular carcinoma cells possess high capacity for tumorigenicity. Int. J. Cancer.

[61] Wright M.H., Calcagno A.M., Salcido C.D., Carlson M.D., Ambudkar S.V., Varticovski L. (2008). Brca1 breast tumors contain distinct CD44+/CD24− and CD133+ cells with cancer stem cell characteristics. Breast Cancer Res..

[62] Brantley D.M., Chen C.L., Muraoka R.S., Bushdid P.B., Bradberry J.L., Kittrell F., Medina D., Matrisian L.M., Kerr L.D., Yull F.E. (2011). Nuclear factor-kappa B (NF-kappa B) regulates proliferation and branching in mouse mammary epithelium. Mol. Biol. Cell.

[63] Demicco E.G., Kavanagh K.T., Romieu-Mourez R., Wang X., Shin S.R., Landesman-Bollag E., Seldin D.C., Sonenshein G.E. (2005). RelB/p52 NF-kappaB complexes rescue an early delay in mammary gland development in transgenic mice with targeted superrepressor IkappaB-alpha expression and promote carcinogenesis of the mammary gland. Mol. Cell Biol..

[64] Cao Y., Luo J.L., Karin M. (2007). IkappaB kinase alpha kinase activity is required for self-renewal of ErbB2/Her2-transformed mammary tumor-initiating cells. Proc. Natl. Acad. Sci. USA.

[65] Liu R., Wang X., Chen G.Y., Dalerba P., Gurney A., Hoey T., Sherlock G., Lewicki J., Shedden K., Clarke M.F. (2007). The prognostic role of a gene signature from tumorigenic breast-cancer cells. N. Engl. J. Med..

[66] Lam J.S., Reiter R.E. (2006). Stem cells in prostate and prostate cancer development. Urol. Oncol..

[67] Klonisch T., Wiechec E., Hombach-Klonisch S., Ande S.R., Wesselborg S., Schulze-Osthoff K., Los M. (2008). Cancer Stem cell markers in common cancers-therapeutic implications. Trends Mol. Med..

[68] Takaishi S., Okumura T., Tu S., Wang S.W., Shibata W., Vigneshwaran R., Gordon S.A.K., Shimada Y., Wang T.C. (2009). Identification of gastric cancer stem cells using the cell surface marker CD44. Stem Cells.

[69] Jang Y.Y., Sharkis S.J. (2007). A low level of reactive oxygen species selects for primitive hematopoietic stem cells that may reside in the low-oxygenic niche. Blood.

[70] Ito K., Hirao A., Arai F., Matsuoka S., Takubo K., Hamaguchi I., Nomiyama K., Hosokawa K., Sakurada K., Nakagata N., Ikeda Y., Mak T.W., Suda T. (2004). Regulation of oxidative stress by ATM is required for self-renewal of haematopoietic stem cells. Nature.

[71] Diehn M., Cho R.W., Lobo N.A., Kalisky T., Dorie M.J., Kulp A.N., Qian D., Jessica S. Lam J.S., Ailles L.E., Wong M., Joshua B., Kaplan M.J., Wapnir I., Dirbas F.M., Somlo G., Garberoglio C., Paz B., Shen J., Lau S.K., Stephen R. Quake S.R., Brown J.M., Weissman I.L., Clarke M.F. (2009). Association of reactive oxygen species levels and radioresistance in cancer stem cells. Nature.

[72] Szatrowski T.P., Nathan C.F. (1991). Production of large amounts of hydrogen peroxide by human tumor cells. Cancer Res..

[73] Kawanishi S., Hiraku Y., Pinlaor S., Ma N. (2006). Oxidative and nitrative DNA damage in animals and patients with inflammatory diseases in relation to inflammation-related carcinogenesis. Biol. Chem..

[74] Zhou Y., Hileman E.O., Plunkett W., Keating M.J., Huang P. (2003). Free radical stress in chronic lymphocytic leukemia cells and its role in cellular sensitivity to ROS generating anticancer agents. Blood.

[75] Kamiguti A.S, Serrander L., Lin K., Harris R.J., Cawley J.C., Allsup D.J., Slupsky J.R., Kraus K.H., Zuzel M. (2005). Expression and activity of NOX5 in the circulating malignant B cells of hairy cell leukemia. J. Immunol..

[76] Oberley T.D., Oberley L.W. (1997). Antioxidant enzyme levels in cancer. Histol. Histopathol..

[77] Hu Y., Rosen D.G., Zhou Y., Feng L., Yang G., Liu J. L., Huang P. (2005). Mitochondrial manganese-superoxide dismutase expression in ovarian cancer: role in cell proliferation and response to oxidative stress. J. Biol. Chem..

[78] Saydama N., Kirb A., Demirb Ö., Hazanc E., Otoc Ö, Saydama O., Güner G.  (1997). Determination of glutathione, glutathione reductase, glutathione peroxidase and glutathione S-transferase levels in human lung cancer tissues. Cancer.

[79] Townsend D.M., Tew K.D. (2003). The role of glutathione-S-transferase in anti-cancer drug resistance. Oncogene.

[80] Yadav S., Zajac E., Singhal S.S., Awasthi S. (2007). Linking stress-signaling, glutathione metabolism, signaling pathways and xenobiotic transporter. Cancer Metastasis Rev..

[81] Trachootham D., Zhang H., Zhang W., Feng L., Du M., Zhou Y., Chen Z., Pelicano H., Plunkett W., Wierda W.G., Keating M.J., Huang P. (2008). Effective elimination of fludarabine-resistant CLL cells by PEITC through a redox-mediated mechanism. Blood.

[82] Zhang H., Trachootham D., Lu W., Carew J., Giles F.J., Keating M.J., Arlinghaus R.B., Huang P. (2008). Effective killing of Gleevec-resistant CML cells with T315I mutation by a natural compound PEITC through redox-mediated mechanism. Leukemia.

[83] Naka K., Muraguchi T., Hoshii T., Hirao A. (2008). Regulation of reactive oxygen species and genomic stability in hematopoietic stem cells. Antioxid.Redox Signal..

[84] Toyokuni S. (2006). Novel aspects of oxidative stress associated carcinogenesis. Antioxid. Redox Signal..

[85] Ghaffari S. (2008). Oxidative stress in the regulation of normal and neoplastic hematopoiesis. Antioxid. RedoxSignal..

[86] Lockshin R.A., Zakeri Z. (2004). Apoptosis, autophagy, and more. Inter. J. Biochem. Cell Biol..

[87] Schwartzman R.A., Cidlowski L.A. (1993). Apoptosis: the biochemistry and molecular biology of programmed cell death. Endocrine Rev..

[88] Ashkenazi A., Dixit V.M. (1998). Death receptors: signaling and modulation. Science.

[89] Gorman A., McGowan A., Cotter T.G. (1997). Role of peroxide and superoxide anion during tumour cell apoptosis. FEBSLett..

[90] Meyer M., Schreck R., Baeuerle P.A. (2005). H^2^O^2^ and antioxidants have opposite effects on activation of NF-kappa B and AP-1 in intact cells: AP-1 as secondary anti-oxidant responsive factor. EMBO J..

[91] Gottlieb E., Vander Heiden M.G., Thompson C.B. (2000). Bcl-x(L) prevents the initial decrease in mitochondrial membrane potential and subsequent reactive oxygen species production during tumor necrosis factor alpha-induced apoptosis. Mol. Cell Biol..

[92] Price R., Vugt M.V., Nosten F., Luxemburger C., Brockman A., Phaipun L., Chongsuphajaisiddhi T., White N. (1998). Artesunate *versus* artemether for the treatment of recrudescent multidrugresistant Plasmodium falciparum malaria. Am. J. Trop. Med..

[93] Ribeiro I.R., Ollario P. (1998). Safety of artemisinin and its derivatives. A review of published and unpublished clinical trials. Med. Trop. (Mars).

[94] Adjuik M., Babiker A., Garner P., Olliato P., Taylor W., White N. (2004). International Artemisinin Study Group. Artesunate combinations for treatment of malaria: meta-analysis. Lancet.

[95] Efferth T. (2006). Molecular pharmacology and pharmacogenomics of artemisinin and its derivatives in cancer cells. Curr. Drug Targets.

[96] Schulze-Bergkamen H., Krar P.H. (2004). Apoptosis in cancer–implications for therapy. Semin. Oncol..

[97] Debatin K.M., Krammer P.H. (2004). Death receptors in chemotherapy and cancer. Oncogene.

[98] Gordi T., Lepist E.I. (2004). Artemisinin derivatives: toxic for laboratory animals, safe for humans?. Toxicol. Lett..

[99] Dell’Eva R., Pfeffer U., Vene R., Anfosso L., Forlani A., Albini A., Efferth T. (2004). Inhibition of angiogenesis *in vivo* and growth of Kaposi’s sarcoma xenograft tumors by the anti-malarial artesunate. Biochem. Pharmacol..

[100] Orrenius S. (2004). Mitochondrial regulation of apoptotic cell death. Toxicol. Lett..

[101] Muller I., Niethammer D., Bruchelt G. (1998). Anthracycline-derived chemotherapeutics in apoptosis and free radical Cytotoxicity. Int. J. Mol. Med..

[102] Efferth T., Giaisi M., Merling A., Krammer P.H., Li-Weber M. (2007). Artesunate Induces ROS-Mediated Apoptosis in Doxorubicin-Resistant T Leukemia Cells. PLoS ONE.

[103] Eskelinen E.L. (2008). New insights into the mechanisms of macroautophagy in Mammalian cells. Inter. Rev.Cell Mol. Biol..

[104] Galluzzi L., Maiuri M.C., Vitale I., Zischka H., Castedo M., Zitvogel L., Kroemer G. (2007). Cell death modalities: classification and pathophysiological implications. Cell Death Differ..

[105] Kihara A., Noda T., Ishihara N., Ohsumi Y. (2001). Two distinct vps34 phosphatidylinositol-3-kinase complexes function in autophagy and carboxypeptidase Y sorting in Saccharomyces cerevisiae. J. Cell Biol..

[106] Ohsumi Y. (2001). Molecular dissection of autophagy: two ubiquitin-like systems. Nat. Rev. Mol. Cell Biol..

[107] Shintani T., Klionsky D.J. (2004). Autophagy in Health and Disease: A Double-Edged Sword. Science.

[108] Cuervo A.M. (2004). Autophagy: In sickness and in health. Trends Cell Biol..

[109] Levine B. (2005). Eating Oneself and Uninvited Guests: Minireview Autophagy-Related Pathways in Cellular Defense. Cell.

[110] Tu B.P., Weissman J.S. (2004). Oxidative protein folding in eukaryotes: mechanisms and consequences. J. Cell Biol..

[111] Fariss M.W., Chan C.B., Patel M., Van Houten B., Orrenius S. (2005). Role of mitochondria in toxic oxidative stress. Mol. Inter..

[112] Wang S.H., Shih Y.L., Ko W.C., Wei Y.H., Shih C.M. (2008). Cadmium-induced autophagy and apoptosis are mediated by a calcium signaling pathway. Cell. Mol. Life Sci..

[113] Wang S.H., Shih Y.L., Kuo T.C, Ko W.C., Shih C.M. (2009). Cadmium Toxicity toward Autophagy through ROS-Activated GSK-3b in Mesangial Cells. Toxicol. Sci..

[114] Gozuacik D., Kimchi A. (2004). Autophagy as a cell death and tumor suppressor mechanism. Oncogene.

[115] Levine B., Yuan J. (2005). Autophagy in cell death: an innocent convict?. J. Clin. Invest..

[116] Li N., Ragheb K., Lawler G., Sturgis J., Rajwa B., Melendez J.A., Robinson J.P. (2003). Mitochondrial complex I inhibitor rotenone induces apoptosis through enhancing mitochondrial reactive oxygen species production. J. Biol. Chem..

[117] Pelicano H., Carney D., Huang P. (2004). ROS stress in cancer cells and therapeutic implications. Drug Resist. Updat..

[118] Scherz-Shouval R., Shvets E., Fass E., Shorer H., Gil L., Elazar Z. (2007). Reactive oxygen species are essential for autophagy and specifically regulate the activity of Atg4. EMBO J..

[119] Yu L., Wan F., Dutta S., Welsh S., Liu Z., Freundt E., Baehrecke E.H., Lenardo M. (2006). Autophagic programmed cell death by selective catalase degradation. Proc. Natl. Acad. Sci. USA.

[120] Reynolds B.A., Tetzlaff W., Weiss S. (1992). A multipotent EGF-responsive striatal embryonic progenitor cell produces neurons and astrocytes. J. Neurosci..

[121] Jacks T., Weinberg R.A. (1996). Cell-cycle control and its watchman. Nature.

[122] Sugimura T. (1998). A new concept of co-mutagenicity from a phenomenon forgotten for the past two decades: Is it more important than previously expected?. Environ. Health Perspect..

[123] Norman J., Cristina Cellurale K., Davis R.J. (2007). A Radical Role for p38 MAPK in Tumor Initiation. Cancer Cell.

[124] Martindale J.L., Holbrook N.J. (2002). Cellular response to oxidative stress: signaling for suicide and survival. J. Cell. Physiol..

[125] Chatterjee S., Fisher A.B. (2004). ROS to the rescue. Am. J. Physiol. Lung Cell Mol. Physiol..

[126] Kurata S. (2000). Selective activation of p38 MAPK cascade and mitotic arrest caused by low level oxidative stress. J. Biol. Chem..

[127] Marshall H.E., Hess D.T., Stamler J.S. (2004). S-Nitrosylation: Physiological regulation of NF-kappaB. Proc. Natl. Acad. Sci. USA.

[128] Xia Z., Dickens M., Raingeaud J., Davis R.J., Greenberg M.E. (1995). Opposing effects of ERK and JNK-p38MAPkinases on apoptosis. Science.

[129] Wang D., Kreutzer D.A., Esigmann J.M. (1998). Mutagenicity and repair of oxidative DNA damage: insights from studies using defined lesions. Mutat. Res..

[130] Bours V., Bentires-Alj M., Hellin A-C., Viatour P., Robe P., Delhalle S., Benoit V., Merville M-P. (1999). Nuclear factor-κB, cancer, and apoptosis. Biochem. Pharmacol..

[131] Kennedy N.J., Davis R.J. (2003). Role of JNK in tumor development. Cell Cycle.

[132] Bulavin D.V., Phillips C., Nannenga B., Timofeev O., Donehower L.A., Anderson C.W., Appella E., Fornace A.J. (2004). Inactivation of the Wip1 phosphatase inhibits mammary tumorigenesis through p38 MAPK-mediated activation of the p16(Ink4a)-p19(Arf) pathway. Nat. Genet..

[133] Ivanova N.B., Dimos J.T., Schaniel C., Hackney J.A., Moore K.A., Lemischka I.R. (2002). A stem cell molecular signature. Science.

[134] Kunduzova O.R., Bianchi P., Pizzinat N., Escourrou G., Seguelas M.H., Parini A., Cambon C. (2002). Regulation of JNK/ERK activation, cell apoptosis, and tissue regeneration by monoamine oxidases after renal ischemia-reperfusion. FASEB J..

[135] Morel A.P., Marjory Lievre M., Thomas C., Hinkal G., Ansieau S., Alain Puisieux A. (2008). Generation of Breast Cancer Stem Cells through Epithelial-Mesenchymal Transition. PLoS ONE.

[136] Das B., Tsuchida R., Baruchel S., Malkin D., Yeger H. The role of VEGF/Flt1 signaling in the maintenance of neuroblastoma side-population celll “stemness” during hypoxia. Advances in Neuroblastoma Research meeting.

[137] Das B. (2007). The role of VEGF autocrine signaling in hypoxia and oxidative stress driven “stemness switch”: implications in solid tumor progression and metastasis. PhD thesis.

[138] Tsuchida R., Das B., Yeger H., Koren G., Shibuya M., Thorner P.S., Baruchel S., Malkin D. (2008). Cisplatin treatment increases survival and expansion of a highly tumorigenic side-population fraction by upregulating VEGF/Flt1 autocrine signaling. Oncogene.

[139] Kunath T., Saba-El-Leil M., Almousailleakh M., Wray J., Meloche S., Smith A. (2007). FGF stimulation of the Erk1/2 signalling cascade triggers transition of pluripotent embryonic stem cells from self-renewal to lineage commitment. Development.

[140] Ito K., Hirao A., Arai F., Takubo K., Matsuoka S., Miyamoto K., Ohmura M., Naka K., Hosokawa K., Ikeda Y., Suda T. (2006). Reactive oxygen species act through p38 MAPK to limit the lifespan of hematopoietic stem cells. Nat. Med..

[141] Bonnet D., Dick J.E. (1997). Human acute myeloid leukemia is organized as a hierarchy that originates from a primitive hematopoietic cell. Nat. Med..

[142] Cook J.A., Giu D., Wink D.A., Krishna M.C., Russo A., Mitchell J.B. (2004). Oxidative stress, redox, and the tumor microenvironment. Semin. Radiat. Oncol..

[143] Azad N., Rojanasakul Y., Vallyathan V. (2008). Inflammation and lung cancer: Roles of reactive oxygen/nitrogen species. J. Toxicol. Environ. Health B.

[144] Dobrovolskaia M.A., Kozlov S.V. (2005). Inflammation and cancer: when NF-kappaB amalgamates the perilous partnership. Curr. Cancer Drug Targets.

[145] Karin M. (2006). Nuclear factor-κB in cancer development and progression. Nature.

[146] Drost J., Agami R. (2009). Transformation Locked in a Loop. Cell.

[147] Klaunig J.E., Kamendulis L.M. (2004). The Role of Oxidative Stress in Carcinogenesis. Ann. Rev. Pharmacol. Toxicol..

[148] Kaltschmidt B., Kaltschmidt C., Beyaert R. (2003). NF-kB in the nervous system.

[149] Denk A., Wirth T., Baumann B. (2000). NF-kappaB transcription factors: critical regulators of hematopoiesis and neuronal survival. Cytokine Growth Factor Rev..

[150] Qiu P., Pan P.C., Govind S. (1998). A role for the Drosophila Toll/Cactus pathway in larval hematopoiesis. Development.

[151] Ghosh S., May M.J., Kopp E.B. (1998). NF-kappa B and Rel proteins: evolutionarily conserved mediators of immune responses. Annu. Rev. Immunol..

[152] Liou H.C., Baltimore D. (1993). Regulation of the NF-kappa B/rel transcription factor and I kappa B inhibitor system. Curr. Opin. Cell Biol..

[153] Kang H.B., Kim Y.E., Kwon H.J., Sok D.E., Lee Y. (2007). Enhancement of NF kappaB expression and activity upon differentiation of human embryonic stem cell line SNUhES3. Stem Cells Dev..

[154] Molofsky A.V., Pardal R., Iwashita T., Park I.K., Clarke M.F., Morrison S. J. (2003). Bmi-1 dependence distinguishes neural stem cell self-renewal from progenitor proliferation. Nature.

[155] Lessard J., Sauvageau G. (2003a). Bmi-1 determines the proliferative capacity of normal and leukaemic stem cells. Nature.

[156] Park I.K., Qian D., Kiel M., Becker M.W., Pihalja M., Weissman I.L., Morrison S.J., Clarke M.F. (2003). Bmi-1 is required for maintenance of adult self-renewing haematopoietic stem cells. Nature.

[157] Sawa M., Yamamoto K., Yokozawa T., Kiyoi H., Hishida A., Kajiguchi T., Seto M., Kohno A., Kitamura K., Itoh Y., Asou N., Hamajima N., Emi N., Naoe T. (2005). BMI-1 is highly expressed in M0-subtype acute myeloid leukemia. Int. J. Hematol..

[158] Androutsellis-Theotokis A., Leker R.R., Soldner F., Hoeppner D.J., Ravin R., Poser S.W., Rueger M.A., Bae S.K., Kittappa R., McKay R.D.G. (2006). Notch signalling regulates stem cell numbers *in vitro* and *in vivo*. Nature.

[159] Lobo N.A., Shimono Y., Qian D., Clarke M. F. (2007). The Biology of Cancer Stem Cells. Annu. Rev. Cell Dev. Biol..

[160] Taipale J., Beachy P.A. (2001). The Hedgehog and Wnt signaling pathways in cancer. Nature.

[161] Reya T., Duncan A.W., Ailles L., Domen J., Scherer D.C., Willert K., Hintz L., Nusse R., Weissman I.L. (2003). A role for Wnt signaling in self-renewal of hematopoietic stem cells. Nature.

[162] Reguart N., He B., Taron M., You L., Jablons D.M., Rosell R. (2005). The role of Wnt signaling in cancer and stem cells. Fut. Oncol..

[163] Zhang J., Grindley J.C., Yin T., Jayasinghe S., He X.C., Ross J.T., Haug J.S., Rupp D., Porter-Westpfahl K.S., Wiedemann L.M., Wu H., Li L. (2006). PTEN maintains haematopoietic stem cells and acts in lineage choice and leukaemia prevention. Nature.

[164] Chow L.M., Baker S.J. (2006). PTEN function in normal and neoplastic growth. Cancer Lett..

[165] Leung C., Lingbeek M., Shakhova O., Liu J., Tanger E., Saremaslani P., Van Lohuizen M., Marino S. (2004). Bmi1 is essential for cerebellar development and is overexpressed in human medulloblastomas. Nature.

[166] Athar M., Tang X., Lee J.L., Kopelovich L., Kim A.L. (2006). Hedgehog signalling in skin development and cancer. Exp. Dermatol..

[167] Palma V., Lim D.A., Dahmane N., Sanchez P., Brionne T.C., Herzberg C.D., Gitton Y., Carleton A., Alvarez-Buylla A., Ruiz i Altaba A. (2005). Sonic hedgehog controls stem cell behavior in the postnatal and adult brain. Development.

[168] Ruizi Altaba A., Sanchez P., Dahmane N. (2002). Gli and hedgehog in cancer: tumours, embryos and stem cells. Nat. Rev. Cancer.

[169] Roche J., Zeng C., Barón A, Gadgil S., Gemmill R.M., Tigaud I., Thomas X., Drabkin H.A. (2004). Hox expression in AML identifies a distinct subset of patients with intermediate cytogenetics. Leukemia.

